# Assessment of SAR in Road-Users from 5G-V2X Vehicular Connectivity Based on Computational Simulations

**DOI:** 10.3390/s22176564

**Published:** 2022-08-31

**Authors:** Marta Bonato, Gabriella Tognola, Martina Benini, Silvia Gallucci, Emma Chiaramello, Serena Fiocchi, Marta Parazzini

**Affiliations:** 1 Institute of Electronics, Computer and Telecommunication Engineering (IEIIT), CNR, 20133 Milano, Italy; 2 Department of Electronics, Information and Bioengineering (DEIB), Politecnico di Milano, 20133 Milano, Italy

**Keywords:** 5G-V2X antenna, Cooperative Intelligent Transport System, RF human exposure, SAR assessment, computational method

## Abstract

(1) Background: Cooperative Intelligent Transportation Systems (C-ITS) will soon operate using 5G New-Radio (NR) wireless communication, overcoming the limitations of the current V2X (Vehicle-to-Everything) wireless communication technologies and increasing road-safety and driving efficiency. These innovations will also change the RF exposure levels of pedestrians and road-users in general. These people, in fact, will be exposed to additional RF sources coming from nearby cars and from the infrastructure. Therefore, an exposure assessment of people in the proximity of a connected car is necessary and urgent. (2) Methods: Two array antennas for 5G-V2X communication at 3.5 GHz were modelled and mounted on a realistic 3D car model for evaluating the exposure levels of a human model representing people on the road near the car. Computational simulations were conducted using the FDTD solver implemented in the Sim4Life platform; different positions and orientations between the car and the human model were assessed. The analyzed quantities were the Specific Absorption Rate on the whole body (SAR_wb_), averaged over 10 g (SAR_10g_) in specific tissues, as indicated in the ICNIRP guidelines. (3) Results: the data showed that the highest exposure levels were obtained mostly in the head area of the human model, with the highest peak obtained in the configuration where the main beam of the 5G-V2X antennas was more direct towards the human model. Moreover, in all configurations, the dose absorbed by a pedestrian was well below the ICNIRP guidelines to avoid harmful effects. (4) Conclusions: This work is the first study on human exposure assessment in a 5G-V2X scenario, and it expands the knowledge about the exposure levels for the forthcoming use of 5G in connected vehicles.

## 1. Introduction

In the recent years, the automotive field has been experiencing fast and pervasive technological innovation towards the new concept of Cooperative Intelligent Transportation Systems (C-ITS) mobility [1]. C-ITS embraces a wide variety of communications-related applications intended to increase travel safety, minimize environmental impacts, improve transport efficiency and maximize the social and economic benefits of transportation to both commercial users and the general public. C-ITS is primarily based on wireless communication technologies for vehicular communication, including vehicle-to-vehicle (V2V), vehicle-to-infrastructure (V2I), vehicle-to-network (V2N) and vehicle-to-pedestrian (V2P) communications. All these types of communications belong to the generic umbrella of vehicle-to-everything (V2X) communications [2,3]. The aim of vehicular communication is to make transportation more enjoyable, safer and greener thanks to the establishment of the so-called ‘network of vehicles’, i.e., a network of vehicles that communicate with road infrastructure, road users and mobile infrastructure to monitor and manage traffic congestions, to prevent collisions and incidents and to share real-time traffic information and alert signals. Finally, forthcoming autonomous driving vehicles will heavily rely on vehicular communication technology [4,5]. Currently, V2X communication is based on direct communication and network communication. Direct communication provides ad hoc communication links for V2V and V2I communications and works at the ITS 5.9 GHz band using the IEEE 802.11p standard protocol in the US and the ITS-G5 protocol in the European C-ITS initiative [6,7]. Network communication, also named Cellular-V2X (C-V2X) communication, is used for short-range V2V, V2I and V2P use cases in the unlicensed ITS 5.9 GHz band using the IEEE802.11p protocol but also for handling wide-area infrastructure-based communications (V2N) use cases (e.g., infotainment and latency-tolerant road safety messages, traffic conditions alerts, etc.) using the regular band licensed for cellular networks (e.g., the 3G/4G and LTE band) [8,9].

In addition, C-V2X will also soon be operated through the 5G New-Radio (NR) wireless communication [10,11,12]. The combination of 5G candidate frequency bands in the low-middle- and high-band ranges will enable technology improvements and services, overcoming the limitations in communication speeds, latency and the available bandwidth of the current cellular technologies (e.g., 3GPP LTE and IEEE 802.11p) and thus increasing road-safety and driving efficiency. Indeed, the use of 5G NR communication will provide both a wider coverage area and a higher system capacity never reached before, such as end-to-end latencies below 5 ms, ultra-high reliability close to 99.999%, positioning accuracy down to 5 cm and the handling of a very large number of connected vehicles, necessary in the future V2X communications and C-ITS mobility world [4,13]. The standardization of the 5G-V2X protocol for vehicular communication is still under discussion by the 3rd Generation Partnership Project (3GPP) consortium. In detail, 5G-V2X will work not only in the ITS unlicensed band at 5.9 GHz but also in two additional larger frequency ranges, namely, frequency range 1 (FR1), from 410 kHz to 7.125 GHz, and frequency range 2 (FR2), from 24.25 to 52.6 GHz, in the millimeter waves spectrum, to satisfy the new technology requirements for low latency and ultra-high reliability communications [11,12].

5G-V2X communication will be possible thanks to the forthcoming deployment of 5G network infrastructure and specific 5G-V2X antennas for the car. Efforts have been made recently to design and evaluate the performance and characteristics of these forthcoming 5G-V2X car antennas (see, e.g., [14,15,16,17,18,19,20]). One current topic of research is the design of 5G-V2X antennas with a small size, high gain and broad beam-width coverage for this type of vehicular communications [14]. Some studies deal with the characterization of multi-band antennas with different form factors and integrated communication standards (i.e., LTE/4G, 5G and radars) [15,16,17]. Others focused on the study and adoption of antenna arrays in both of the new 5G frequency ranges (i.e., FR1 and FR2), which represents the most suitable solution to increase gain and overcome the path loss problem [14,18,19,20].

The introduction of 5G-V2X vehicular communication will have an impact not only on novel transportation and mobility services but also on the users (i.e., drivers and passengers) and people in proximity (e.g., pedestrians and road-users in general) of the connected car: as a matter of fact, these people will be exposed to additional and not yet thoroughly investigated RF sources coming from their own car, from nearby cars and from the infrastructure. The assessment of possible modifications in human RF exposure levels due to the new 5G wireless communications is a timely issue, as evidenced in the Technical Information Statement (TIS) of the Committee on Man and Radiation (COMAR), which addresses health and safety issues concerning the exposure of the general public to radiofrequency (RF) fields from 5G [21].

One way to characterize these human RF-EMF exposure changes is represented by monitoring the dose of RF-EMF absorbed in tissues and organs using computational simulations [22,23]. As for 5G exposure assessment based on computational methods, up until now, efforts have been made to assess human exposure to plane waves, dipoles and array antennas operated at the FR1 and FR2 ranges both in near-field and far-field conditions in mobile network communication scenarios [24,25,26,27,28,29,30].

To the best of the authors’ knowledge, no computational studies have addressed the specific 5G-V2X communication scenario and its peculiar characteristics, i.e., the use of FR1 and FR2 bands and the use of novel 5G-V2X array antennas with beamforming capability. As described in a recent literature survey [31] on RF human exposure in the connected car due to V2X, car sensing and intra-vehicle wireless communication, the majority of previous computational studies investigated the exposure levels of drivers and passengers from generic personal wireless communication technologies used inside the vehicle, such as mobile phones, Bluetooth and WiFi devices [32,33,34]. Only a few studies assessed the field exposure generated by antennas working at frequencies used in V2X communications [35,36,37,38]. For examples, in the study of Baramili et al. [35], the EMF distributions at the driver seat were evaluated for various positions of a multiband 4G/5G antenna that can be easily integrated into the front and rear glasses of vehicles; in the first paper of Ruddle et al. [36], the levels of the specific absorption rate, that is, the power absorbed per unit mass of the body, for homogeneous models in driver and passenger positions were evaluated considering car antennas external quarter-wave monopoles at 400 MHz and 900 MHz, with an input power of 1 W, showing exposure values well below the recommended limits [39]. In the second paper of Ruddle et al. [37], a preliminary estimation was conducted about the possible contributions in terms of exposure from a number of typical automotive systems based on different wireless communications technologies (i.e., 5.8 GHz antenna, mobile phone, Wi-Fi, Bluetooth, radar systems), showing that the number of sources present in the automotive environment could produce sufficiently high individual exposure levels that should be monitored. Finally, in the paper of Tognola et al. [38], the exposure of a driver in a connected car equipped with four V2V antennas mounted on the car roof was assessed in terms of the specific absorption rate. The antennas were operated at the standard V2V communication frequency in the ITS 5.9 GHz band, according to the IEEE 802.11p wireless protocol for mobility, with an input power of 30 W; the results obtained were again well below the basic restrictions for RF exposure for the general public [39].

None of these previous studies were focused on the assessment of RF exposure in the specific 5G-V2X exposure scenario. In the present study, we explore, using computational simulations based on the FDTD (Finite-Difference Time-Domain) method, the RF exposure generated by vehicular 5G-V2X communication by assessing the exposure levels for a person (e.g., a road user) in near proximity to a car equipped with two 5G-V2X antennas at 3.5 GHz, in the FR1 frequency range. We decided to evaluate the exposure levels of pedestrians since the two 5G-V2X antennas were mounted externally on the car body and the main beam is directed primarily towards the surrounding environment. Exposure levels were calculated in a realistic and detailed 3D human body model using a computational approach and were expressed in terms of the specific absorption rate mediated over the entire body (SAR_wb_) and over 10 g (SAR_10g_) in selected tissues, as specified in the ICNIRP guidelines [39].

## 2. Materials and Methods

The present section illustrates the details of the car model, the 5G-V2X antenna model, the human model, the simulated exposure scenario and the procedure used for assessing RF exposure.

### 2.1. The Car Model

**Figure 1:**
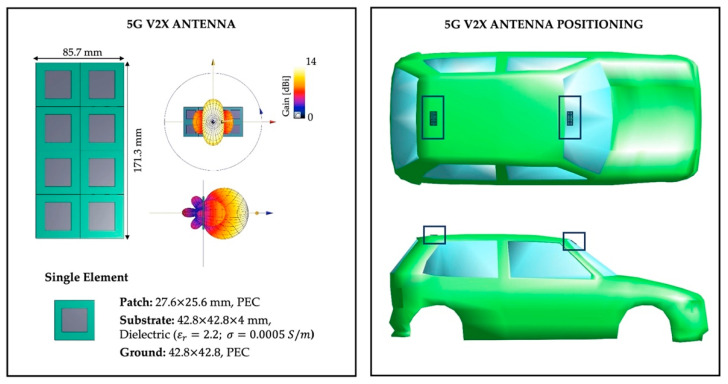
On the **left**, the characteristics and geometry of the 5G-V2X antenna. On the **right**, the positions of the two 5G-V2X antennas on the car.

For the car, as can be seen in Figure 1, we use a model resembling, in terms of dimension and shape, a typical city car. In detail, the model consists of the car body, modeled as PEC material, and six windows characterized by glass material (density ρ=2500 kg/m^3^; conductivity σ=0.0025 S/m; relative permittivity $\varepsilon_{r}=2.6$). Lastly, the interior of the car was filled by air because it was demonstrated that the electric field generated in the car by external or internal sources is only marginally affected by the materials typically used in car interiors, such as foam and thin plastics [40].

### 2.2. The 5G-V2X Antenna Model and Positioning

Two 5G-V2X antennas at the working frequency of 3.5 GHz were modeled according to the technical specifications indicated by the H2020 5G PPP 5G Communication Automotive Research and innovation (5GCAR) Project [41,42]. Briefly, the 5GCAR Project aimed at identifying 5G solutions to meet the key performance requirements in the most representative and emerging use cases of V2X communication. The 5G-V2X antenna model and setup identified in the 5GCAR Project are based on the 3GPP Release 16 and Release 17 specifications on the use of 5G-V2X in vehicular connectivity, resulting in Uniform Linear Array antennas with maximum 8 Tx elements operating at 3.5 GHz, ITS 5.9 GHz, 28 GHz and 64 GHz (mmWave band) [11,12].

In this work, the antenna model was simulated using the Sim4Life software platform (www.zmt.swiss, accessed on 18 July 2022), and its dimensions and characteristics are displayed in the left part of Figure 1. In detail, each 5G-V2X antenna is modeled as an array antenna of eight elements disposed in two rows of four elements each. Each antenna element consists of a simple patch antenna with three layers: the ground and patch layers, modelled as PEC materials, and the substrate element, modelled as a dielectric material (dielectric properties:$\;\varepsilon_{r}$ = 2.2 and σ = 0.0005 S/m). In order to have the array antenna resonate at the 5G-V2X frequency of 3.5 GHz, the dimensions of the single element were set equal to 42.8×42.8×4  mm, resulting in a total dimension of the eight-element array antenna of 171.3×85.7×4  mm. Each array antenna is driven with an input power of 1 W, simultaneously exciting the eight elements with a harmonic signal with a phase shift of 0° between the eight elements, as illustrated in Figure 1. The resulting antenna maximum gain is about 14 dBi, with the main lobe of the array antenna in the boresight direction (i.e., perpendicular to the plane of the antenna).

We mounted the two antenna models in two different positions on the car body, following the typical settings used in real implementations of 5G vehicular connection [43,44]. As we can see from the right part of Figure 1, the first 5G-V2X array antenna is positioned on the windshield glass, along the midline of the car, while the second antenna is placed on the roof top, in a posterior position, near the rear window.

### 2.3. The Human Model

To evaluate human exposure levels, we used the CAD Ella model V1.3 from the Virtual Population (https://itis.swiss/virtual-population/virtual-population/overview/, accessed on 18 July 2022). The Ella model is optimized for finite-element modeling and represents an average adult female (age = 26 years old, height = 1.63 m, mass = 57.3 kg, BMI = 21.6 kg/m) [45]. The CAD Ella model has a resolution of 0.5 × 0.5 × 0.5 mm³ throughout the entire body and comprises 76 different tissues, whose dielectric properties at 3.5 GHz were chosen according to the literature data [46,47].

### 2.4. The Simulated Exposure Scenario

As for the V2X scenario, we considered the case of a road user (e.g., a pedestrian) standing in the vicinity of a car equipped with two 5G-V2X antennas. We implemented the computational simulations using the finite-difference time-domain (FDTD) solver of the simulation platform SIM4Life (ZMT Zurich Med Tech AG, Zurich, Switzerland, www.zurichmedtech.com, accessed on 18 July 2022) [48].

**Figure 2:**
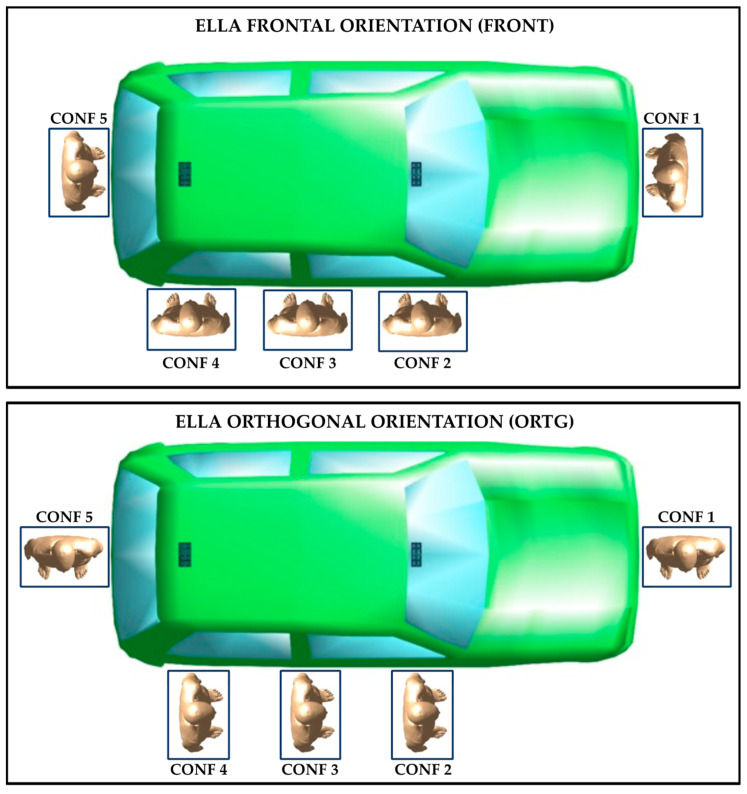
The exposure scenario with the car model, the phantom Ella at the five analyzed configurations and the two antenna arrays (the small dark rectangles at the front and rear of the car). The Ella model is placed in the ‘FRONT’ (**top** panel) and ‘ORTG’ orientation (**bottom** panel) with respect to the car body.

To assess the impact of the position of the phantom on the dose of RF field absorbed, five different configurations of the human model with respect to the car body were considered, as can be seen Figure 2. The chosen configurations are listed in the following:

1. CONF 1: the Ella model is placed next to the frontal car hood, at its midline;
2. CONF 2: the Ella model is placed laterally to the car body, in front of the antenna array mounted on the windscreen;
3. CONF 3: the Ella model is placed laterally to the car body, at an equal distance from the two antennas;
4. CONF 4: the Ella model is placed laterally to the car body, in front of the array antenna mounted on the roof top;
5. CONF 5: the Ella model is placed next to the car trunk, at its midline.

Moreover, for each of the five configurations above, two different orientations—frontal and orthogonal—of the human phantom with respect to the car body were assessed. In the first orientation (i.e., “FRONT” orientation, illustrated in the upper part of Figure 2), the eyes of the model are directed towards the car body, whereas in the second orientation (i.e., “ORTG” orientation, illustrated in the lower part of Figure 2), the Ella model is placed orthogonal to the car body. In each configuration and orientation, the Ella model was placed at the minimum distance from the car model, that is, the outermost point of the human phantom (namely, the toes in the FRONT orientation and the right/left shoulder in the ORTG orientation) was placed 1 mm away from the same line as the outermost point of the car model (namely, the frontal and rear bumpers in CONF1 and CONF5 and the lateral side of the car body in CONF2-4).

For each configuration, the computational domain included the whole human body, the whole car model and the two array antennas. The computational domain was discretized with a nonuniform grid with a maximum step of 1.408 mm for all body tissues, apart from the eye tissues (i.e., cornea, lens, sclera and humor vitreous), which were discretized with a finer grid of 1.043 mm. The total number of discretization cells varied from $857\times 10^{6}$ to $1.102\times 10^{9}$ across configurations. Furthermore, the computational domain was truncated by assuming a perfectly matched layer (PML) absorbing condition at the domain boundaries.

### 2.5. Human Exposure Assessment

To assess human exposure levels, we evaluated, for each configuration, the whole-body Specific Absorption Rate (SAR_wb_) defined as the power absorbed (per kg) over the entire body, the distribution of the Specific Absorption Rate averaged on 10 g of tissue (SAR_10g_) and the peak value of SAR_10g_ (pSAR_10g_). We assessed these quantities for the most superficial tissues (e.g., the skin and eyes) and for the brain tissues of the Ella phantom. For the skin, the SAR_10g_ distributions and pSAR_10g_ values were evaluated at the whole-body and locally at the head, as the two 5G-V2X array antennas are positioned almost at the head level. For the brain, the SAR_10g_ distributions and pSAR_10g_ values were assessed in the whole brain, which included the brain grey and white matter, cerebellum, hippocampus, hypophysis, hypothalamus, medulla oblongata, midbrain, pineal body, pons, thalamus and anterior and posterior commissure. Finally, regarding the eyes, the SAR_10g_ distributions and the pSAR_10g_ values were calculated in the cornea, sclera, lens and humor vitreous tissues. To better characterize the SAR_10g_ distribution within and among tissues across the examined configurations, the quartile coefficient of dispersion (QCD), a unitless measure of the dispersion around the median, was calculated in each examined tissue as follows:

(1) $$\mathrm{QCD}=\frac{\left(Q_{3}-Q_{1}\right)}{\left(Q_{3}+Q_{1}\right)}\;$$

where $Q_{1}$ and $Q_{3}$ are the first and third quartiles of the distribution, respectively. Lastly, the percentage of values higher than 0.7 of the pSAR_10g_ was reported for each tissue to investigate the spread of the SAR around the peak value.

## 3. Results

In this paragraph, we describe the results obtained for the five different configurations evaluated in the frontal and orthogonal orientation of the phantom, in terms of SAR_wb_ and SAR_10g_. Furthermore, we remind readers that all the data and values reported in the present study are obtained by considering an input power of 30 dBm (1 W) for each of the two 5G-V2X antennas, as described in the Materials and Methods section.

**Figure 3:**
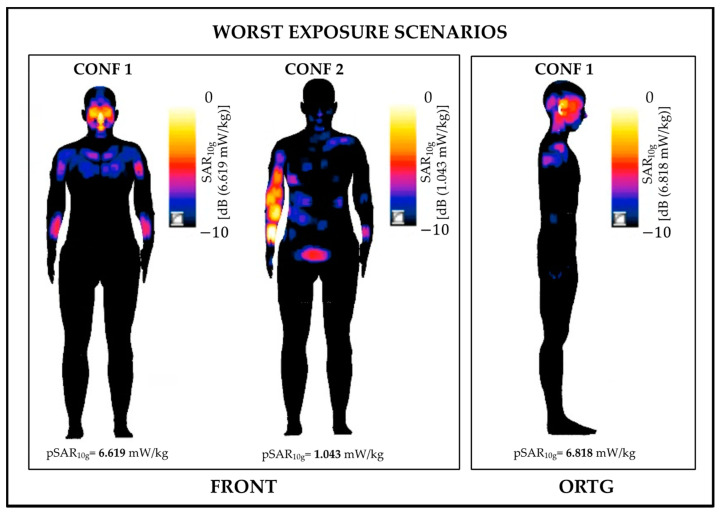
Distributions of SAR_10g_ on the skin tissue for the three cases where pSAR_10g_ was higher than 1 mW/kg. Note that the distributions in the three cases are normalized with respect to their pSAR_10g_.

Figure 3 shows the distribution of SAR_10g_ on the skin. For the sake of clarity, we displayed only the three cases where pSAR_10g_ was higher than 1 mW/kg, namely, CONF 1 in the frontal and orthogonal orientations and CONF2 in the frontal orientation. These three cases correspond to Ella standing closer to the frontal antenna. The high exposure levels found in these three cases are mostly due to the field generated by the front 5G-V2X antenna (mounted on the car windscreen with a tilt angle of 37°), causing the main beam of the 5G-V2X antenna to be somewhat more directed to the phantom.

As seen in Figure 3, in CONF 1, the highest SAR_10g_ values are localized at the head, namely, at the nose and eyes in the frontal orientation and at the ear region in the orthogonal one, whereas the torso and the arms showed lower SAR_10g_ values with respect to the head. In CONF2, in the frontal orientation, the highest exposure levels are localized in the right arm, that is, the body part that is closer to the frontal 5G-V2X antenna.

As a general remark, we further noticed that, in all configurations (included those not shown in Figure 3), the highest SAR_10g_ values were observed in the torso, arms and head area, whereas the legs showed almost negligible values.

As for the exposure at the whole-body, the SAR_wb_ was well below the basic restriction limit of 0.08 W/kg and ranged from 0.001 mW/kg in CONF5 in the frontal orientation to 0.074 mW/kg in CONF1 in the frontal orientation.

**Figure 4:**
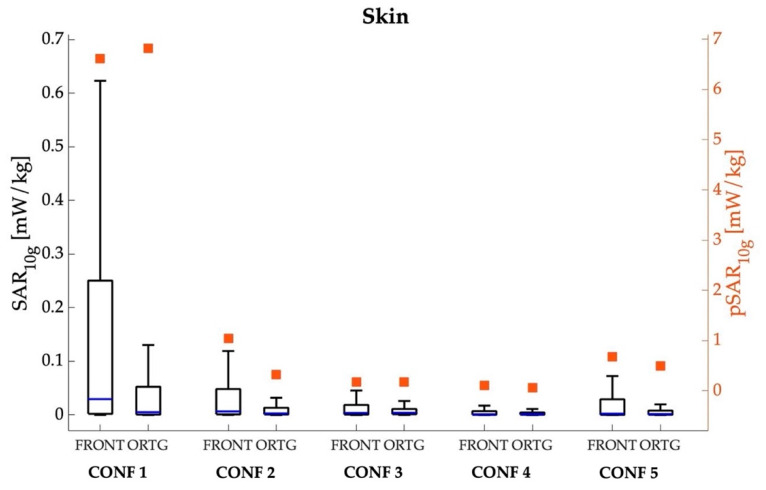
SAR_10g_ (left *y*-axis, box plot) and pSAR_10g_ (right *y*-axis, square symbols) in the skin tissue over the whole body for the five different examined configurations, considering the frontal and orthogonal orientations of the Ella model. The center line in the boxes is the median; the bottom and top edges are the 25th and 75th percentiles. The lower whisker extends to the minimum value, while the upper whisker extends to 1.5 times the height of the box.

**Figure 5:**
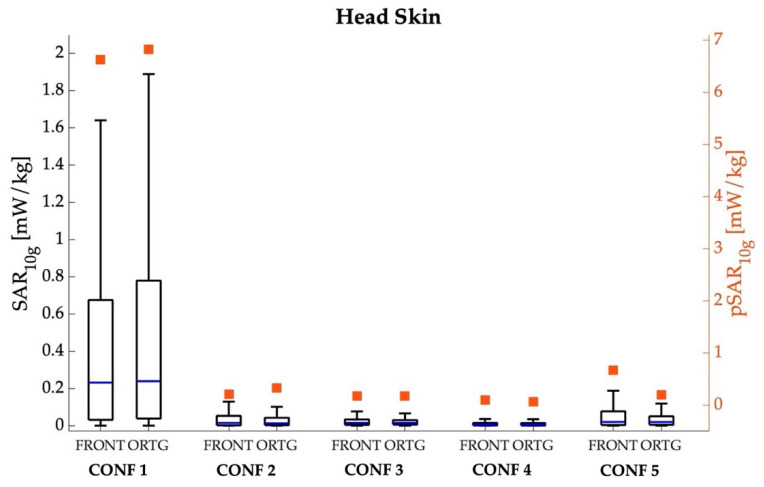
SAR_10g_ (left *y*-axis, box plot) and pSAR_10g_ (right *y*-axis, square symbols) in the skin tissue over the whole body for the five different examined configurations, considering the frontal and orthogonal orientations of the Ella model. The center line in the boxes is the median; the bottom and top edges are the 25th and 75th percentiles. The lower whisker extends to the minimum value, while the upper whisker extends to 1.5 times the height of the box.

**Figure 6:**
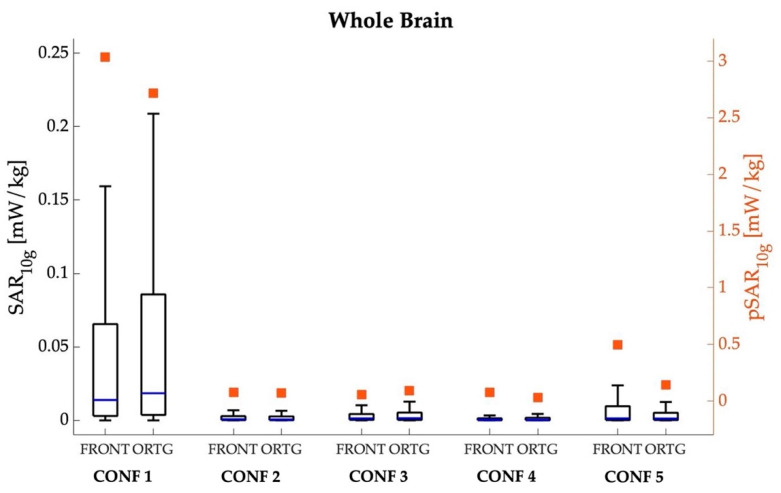
SAR_10g_ (left *y*-axis, box plot) and pSAR_10g_ (right *y*-axis, square symbols) in the brain tissue for the five different examined configurations, considering the frontal and orthogonal orientations of the Ella model. The center line in the boxes is the median; the bottom and top edges are the 25th and 75th percentiles. The lower whisker extends to the minimum value, while the upper whisker extends to 1.5 times the height of the box.

**Figure 7:**
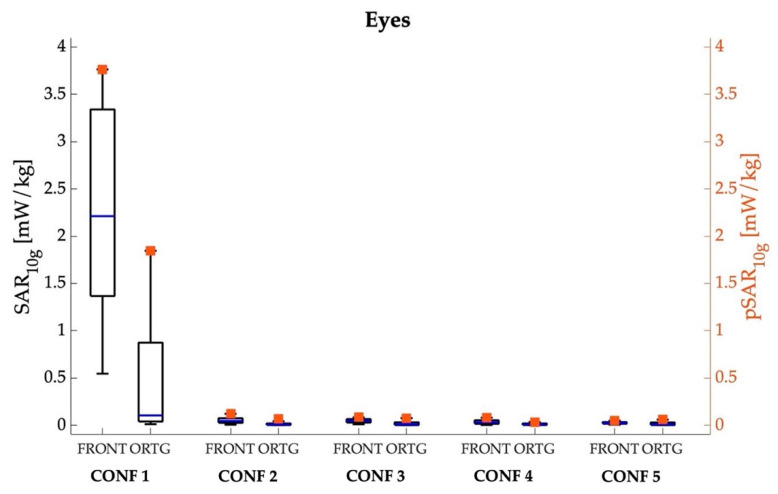
SAR_10g_ (left *y*-axis, box plot) and pSAR_10g_ (right *y*-axis, square symbols) in the eye tissues for the five different examined configurations, considering the frontal and orthogonal orientations of the Ella model. The center line in the boxes is the median; the bottom and top edges are the 25th and 75th percentiles. The lower whisker extends to the minimum value, while the upper whisker extends to 1.5 times the height of the box.

Figure 4, Figure 5, Figure 6 and Figure 7 show the peak value and boxplot of the SAR_10g_ distribution in the skin tissue, head skin tissue, whole brain and eyes, respectively. As a general comment, we notice that the highest pSAR_10g_ values are always obtained in CONF 1 (that is, when Ella is in front of the car hood) for both orientations and all tissues. Moreover, as seen from the boxplot in Figure 4, Figure 5, Figure 6 and Figure 7, for all tissues, configurations and orientations, the distribution of SAR_10g_ exhibits a substantial positive skewness, meaning that SAR_10g_ was mostly distributed towards low exposure levels.

As for the skin over the whole body (Figure 4), the greatest pSAR_10g_ was found in CONF1 and was equal to 6.619 mW/kg (mean = 0.221 mW/kg; median = 0.029 mW/kg) and 6.818 mW/kg (mean = 0.122 mW/kg; median = 0.005 mW/kg) for the frontal and lateral orientations, respectively; the pSAR_10g_ in the other configurations was 84–98% lower than that in CONF1, ranging from 0.061 mW/kg (mean = 0.004 mW/kg; median = 0.001 mW/kg) in CONF4 in the orthogonal orientation to 0.675 mW/kg (mean = 0.037 mW/kg; median = 0.002 mW/kg) in CONF5 in the frontal orientation.

In Figure 5, the same analysis is reported for the skin tissue of only the head. We noticed that the pSAR_10g_ value in the skin of the head was the same as that observed for the skin over the whole body (Figure 4) in all configurations but in CONF 2 in the frontal orientation and CONF 4 in the orthogonal orientation, where the pSAR_10g_ over the skin of the whole body (Figure 4) was higher than at the head. As a matter of fact, in these latter two configurations, the maximum of SAR_10g_ in the skin of the whole body was localized at the arm, because this was the body part closer to the 5G-V2X antennas.

In Figure 6, the analysis is focused on the whole brain, another critical and important region for assessing human exposure levels. As expected, the exposure levels in the brain are lower with respect to the skin in all configurations due to the low penetration of the field radiated by the two 5G-V2X antennas working in the GHz region. The reduction of pSAR_10g_ with respect to the skin ranged from 23% to 92%, depending on the configuration. The distribution of SAR_10g_ in the brain exhibited the same trend observed in the skin of the whole body and of the head, that is, the highest exposure was observed in CONF 1. Namely, the pSAR_10g_ value in CONF 1 is equal to 3.038 mW/kg (mean = 0.066 mW/kg; median = 0.013 mW/kg) for the frontal orientation and 2.716 mW/kg (mean = 0.080 mW/kg; median = 0.002 mW/kg) for the orthogonal one, whereas in the other configurations. the pSAR10g value was 84–99% lower than that in CONF 1.

Lastly, in Figure 7, we analyzed the SAR_10g_ distributions obtained for the eyes. Also for the eyes, the highest exposure levels are obtained in CONF 1 in the frontal orientation, as the antenna beam is more directed to the eyes area than in the orthogonal orientation. The pSAR_10g_ value in CONF 1 is equal to 3.765 mW/kg (mean = 2.280 mW/kg; median = 2.212 mW/kg) for the frontal orientation and 1.845 mW/kg (mean = 0.488 mW/kg; median = 0.103 mW/kg) for the orthogonal one. For the orthogonal orientation, in all the examined configurations, only one eye is much more illuminated by the radiation field of the 5G-V2X antennas, resulting in almost null values of exposure for the eye further away.

Table 1, Table 2, Table 3 and Table 4 list the QCD and the percentage of values higher than 0.7 pSAR_10g_ for the examined tissues.

As for the skin of the whole body (Table 1), the skin of the head (Table 2) and the brain tissues (Table 3), the QCD was high, ranging from 0.689 to 0.987 across tissues and configurations: this is a clear indication of the large dispersion (i.e., large variability) of the SAR_10g_ values over the examined tissues. Within each tissue, the QCD is almost the same among the different configurations, meaning that all configurations are characterized by the same variability of the SAR_10g_ distribution. Additionally, for the tissues in Table 1, Table 2 and Table 3, the percentage of values higher than 0.7 pSAR_10g_ is extremely low, between 0.010% to 5.050%, indicating that the regions where the highest exposure level are localized are extremely narrow.

Finally, as for the eyes (Table 4), the QCD is different between the orthogonal and frontal orientations, it being higher in the orthogonal one. Namely, in the orthogonal orientation, the QCD ranges from 0.760 to 0.914, indicating that the SAR_10g_ distribution is characterized by a high degree of variability (as previously observed in the other tissues). Vice versa, in the frontal orientation, the QCD is significantly lower than that observed in the orthogonal orientation and in the skin, the head and the brain (Table 1, Table 2 and Table 3), ranging from 0.372 to 0.556: this is a clear indication that, in the frontal orientation, the SAR_10g_ distribution is characterized by a low degree of variability and is almost normally distributed. The higher variability observed in the orthogonal orientation is because, in this latter orientation, the exposure is only in one of the two eyes, that is, the one nearer to the antennas. Furthermore, the percentage of values higher than 0.7 pSAR_10g_ in the eye tissues is the highest amongst the investigated tissues, with values between 11.953% and 41.238%, denoting that the highest exposure levels are spread in a large part of the eyes’ tissues, probably due to the small size of the eye tissues compared to the other tissues under examination. For the orthogonal orientation, in all the examined configurations, only one eye is much more illuminated by the radiation field of the antennas, resulting in almost null values of exposure for the eye further away. Furthermore, the percentage of values higher than 0.7 pSAR_10g_ is lower with respect to the frontal orientation, with values from 3.772% to 18.869%, because the highest exposure levels are focused only on one eye.

## 4. Discussion

To the best of the authors’ knowledge, this is the first study on the assessment of human RF exposure in a realistic 5G-V2X communication scenario. Specifically, in the present study, we assessed the dose of RF absorbed by people (e.g., a pedestrian) in the vicinity of a car equipped with two 5G-V2X antennas. To conduct this exposure assessment, first, model of a 5G-V2X antenna was implemented based on the last technical specifications released by 3GPP (Release 16 and 17 [11,12]), resulting in an array antenna of eight elements at the working frequency of 3.5 GHz in the FR1 range for 5G wireless communications. Then, two exemplars of this antenna model were mounted on a 3D realistic model of a city car, according to the typical antenna mounting position, i.e., on the windscreen and on the roof at the back of the car [43,44]. Lastly, the human exposure levels induced by the two antennas were assessed using the Ella model from the Virtual Population by considering different configurations between the car body and the human model to better characterize the impact of the position of the person near the car on the exposure levels. The assessment of the exposure levels was based on the analysis of the SAR_10g_ distribution and pSAR_10g_ values, focusing on the typical tissues of interest for the exposure assessment in the GHz frequency band, which are the skin, the brain and eyes tissues, in line with the analysis conducted in other studies on RF human exposure assessment considering the forthcoming 5G networks deployment [24,25,26,27,28,29,30].

Firstly, and most importantly, in the considered 5G-V2X scenario, the exposure in the different configurations was well below the SAR_10g_ limit of 2 W/kg for the head and torso, as indicated by the ICNIRP guidelines [39]. If the input power of the two 5G-V2X antennas is further scaled to a realistic value of 23 dBm, i.e., 200 mW, as indicated in the technical specification of 3GPP [11,12]), the level of exposure due to a V2X communication scenario is even more significantly reduced.

Our study evidenced that the highest SAR_10g_ values were localized mostly in the head area in both orientations and all examined configurations, except in CONF 2 in the frontal orientation and in CONF 4 in the orthogonal orientation, where pSAR_10g_ was localized in the arm, that is, the part of the body in closer proximity to the 5G-V2X antennas. Furthermore, in all cases, the legs showed values of exposure almost equal to zero. This result was expected because the head and arms are the regions of the human model closest to the 5G-V2X antennas, whereas the legs, being more distant from the antennas, are consequently less exposed.

Moreover, it was noticed that the exposure level depended on the position of the human model near the car. Among all configurations, CONF 1, where Ella stands in front of the car hood, showed the highest pSAR_10g_; in the remaining configurations, pSAR_10g_ was 84% to 98% less than that in CONF 1. This is probably due to the position of the frontal 5G-V2X antenna, it being mounted on the inclined surface of the windshield with a tilt of 37° degrees in the forward direction, thus directing the main beam of radiation more towards the human model than the rear antenna. On the contrary, the rear antenna, being placed on the horizontal plane of the car roof, directs the main beam in the upward direction; consequently, when the human model stands at the back of the car, it is reached almost exclusively by the side lobes of the radiation. The orientation of the human model with respect to the car has only a marginal effect on pSAR_10g_ values.

Regarding CONF 1, for both orientations of the human model, the highest exposure levels are focused on the head area—specifically, at the nose and eyes in the frontal orientation and at the ears in the orthogonal one. This is in line with the study of Uusitupa et al. [49], which evaluated the variation of SAR in 15 human models of different sizes, considering a single-plane wave exposure from 300 to 5000 MHz. This latter study underlined that, under frontal exposure, the typical pSAR_10g_ locations are more in the head/trunk region than in the limbs, particularly in the fingers, toes, nose, ears, chin, penis and testicles regions. This result was because the exposure field can effectively enter inside these body parts which have relatively small dimensions and irregular structures [49].

In all configurations, the highest pSAR_10g_ values were observed in the skin, i.e., the most superficial tissue of the body, whereas in deeper tissues (e.g., the brain), the exposure level was 23% to 92% less than that in the skin. This result is in line with previous studies on RF human exposure assessment that evidenced how the highest peaks of exposure were always localized in the most superficial tissues (i.e., mainly the skin and eyes) because RF-waves penetrate only in the first millimeters of human tissues [24,25,26,27,28,29,30].

For the skin of the whole body and the head and for the brain tissues, the SAR_10g_ distribution was skewed positively, meaning that most of the data were distributed towards the lower range of the exposure level. Additionally, from the analysis of the SAR_10g_ distributions, we discovered that the values of SAR_10g_ higher than 0.7 pSAR_10g_ occupied only small areas over the skin of the whole body and the head and in the whole brain.

Only in the eyes, which have smaller dimensions, SAR_10g_ was more normally distributed, particularly for the frontal orientation of the phantom, and the values higher than 0.7 pSAR_10g_ occupy a wider portion of the eyes, reaching up to 41% of the total volume in the frontal orientation of CONF 1, that is, the configuration where the eyes are most exposed to the field radiated by the windscreen antenna. We found the exact same trend in our previous study that dealt with the RF human exposure assessment in a possible future case of an indoor exposure scenario, where the presence of a 5G access point (AP) was simulated at a working frequency that was almost the same as that in this work (3.7 GHz vs. 3.5 GHz); also, in this study, in the tissues with a smaller extension (e.g., cerebellum and, in particular, the eye tissues), the SAR_10g_ distributions become smoother and less skewed with respect to the SAR_10g_ distributions over the skin on the whole body [25].

Since, in the present paper, a specific 5G-V2X exposure scenario was analyzed for the first time, with its peculiar characteristics (i.e., the use of the FR1 band, the new 5G-V2X array antenna with beamforming capability, the presence of the metallic body of the car), it is difficult to find a fair comparison with previous literature studies. It remains, however, interesting to discuss, at least from a qualitative point of view, similarities and differences with other studies that investigated the RF exposure outside or inside vehicles in the frequency range for mobile communication [32,33,34] and for V2V in the ITS 5.9 GHz band [38]. Table 5 gives an overview, comparing the exposure levels we obtained in terms of SAR from this study with respect to the ones found in these other literature studies [32,33,34,38].

From Table 5, we can notice that, in the studies [32,33,34], different emitting devices (e.g., phones, UMTS, WiMax, Bluetooth devices) were placed inside the vehicle cabin, and the exposure levels were evaluated for the driver and the passengers. The peak SAR found in these studies is much greater than that in our present work, namely, up to 127 W/kg at 1800 MHz in [32], 3.77 W/kg at 900 MHz in [34] and 392.6 mW/kg for a WiMax device in [33], mainly due to the closer distance between the human models and the emitting devices. Furthermore, in the study of Tognola et al. [38], the exposure levels were evaluated in a realistic smart mobility communication scenario operated at 5.9 GHz, where four V2V antennas were modeled as omnidirectional monopoles and placed on a realistic 3D model of a city-car to estimate the SAR levels of a human adult male phantom inside the car. Also in this study, the maximum value of SAR (equal to 1.58 W/kg for the head/torso area) was higher than that found in the present work. However, it is important to notice that, in [38], the input power of each of the four V2X antennas was equal to 30 W (for an input power of 1 W, as in this work, the maximum SAR value became only then equal to 0.05 W/kg), and the distance between the antennas and the phantom was shorter than that in our scenario. Furthermore, interestingly, in the work of Tognola et al. [38], the highest exposure levels were also individuated in the skin tissue and localized only in the body regions closer to the antennas, as in this work. Instead, we found no literature studies that dealt directly with the assessment of the exposure levels of a person in close proximity to the car and not inside. Indeed, to our knowledge, this is the first study about this topic.

## 5. Conclusions

In conclusion, this is first study on human exposure assessment in a forthcoming 5G-V2X scenario. Human exposure levels generated by two 5G-V2X antennas at 3.5 GHz and mounted on a 3D model of a real car were assessed in terms of SAR_10g_ and pSAR_10g_. Different configurations and orientations between the human model and the car body were considered, showing that, in all configurations, the dose absorbed by a road user in the vicinity of the car was well below the basic restrictions for the general public of 0.08 W/kg for whole-body average SAR and of 2 W/kg for the local head and torso SAR, as indicated in the ICNIRP guidelines to avoid harmful effects.

Future studies will deal with the impact of beamforming on human exposure levels and with the combined application of traditional computational methods and machine learning and stochastic techniques to evaluate the variability of these 5G-V2X communication exposure scenarios, not limiting the analysis to a few configurations [50,51].

## Figures and Tables

**Table 1:** pSAR_10g_ of each configuration for the skin tissue.

Configuration	Orientation	QCD	% > 0.7·pSAR_10g_
CONF 1	Frontal	0.984	0.061%
	Orthogonal	0.978	0.053%
CONF 2	Frontal	0.957	0.114%
	Orthogonal	0.878	0.548%
CONF 3	Frontal	0.937	0.036%
	Orthogonal	0.801	0.095%
CONF 4	Frontal	0.953	0.266%
	Orthogonal	0.879	0.173%
CONF 5	Frontal	0.987	0.293%
	Orthogonal	0.926	0.368%

**Table 2:** pSAR_10g_ of each configuration for the skin of the head.

Configuration	Orientation	QCD	% > 0.7·pSAR_10g_
CONF 1	Frontal	0.912	0.456%
	Orthogonal	0.907	0.449%
CONF 2	Frontal	0.931	3.790%
	Orthogonal	0.872	0.970%
CONF 3	Frontal	0.770	0.325%
	Orthogonal	0.689	0.858%
CONF 4	Frontal	0.872	2.382%
	Orthogonal	0.911	0.144%
CONF 5	Frontal	0.917	1.872%
	Orthogonal	0.833	5.050%

**Table 3:** QCD and percentage of values higher than 0.7 pSAR_10g_ of each configuration for the whole brain.

Configuration	Orientation	QCD	% > 0.7·pSAR_10g_
CONF 1	Frontal	0.912	0.026%
	Orthogonal	0.917	0.010%
CONF 2	Frontal	0.879	0.021%
	Orthogonal	0.907	0.023%
CONF 3	Frontal	0.858	0.145%
	Orthogonal	0.893	0.080%
CONF 4	Frontal	0.865	0.022%
	Orthogonal	0.934	0.080%
CONF 5	Frontal	0.957	0.176%
	Orthogonal	0.910	0.044%

**Table 4:** QCD and percentage of values higher than 0.7 pSAR_10g_ of each configuration for the eye tissues ^1^.

Configuration	Orientation	QCD	% > 0.7·pSAR_10g_
CONF 1	Frontal	0.420	41.238%
	Orthogonal	0.914	12.880%
CONF 2	Frontal	0.503	16.170%
	Orthogonal	0.760	3.772%
CONF 3	Frontal	0.398	37.407%
	Orthogonal	0.905	7.189%
CONF 4	Frontal	0.556	22.575%
	Orthogonal	0.803	18.869%
CONF 5	Frontal	0.372	11.953%
	Orthogonal	0.845	15.344%

^1^ Please note that all values reported in the table are computed by considering the SAR_10g_ distribution of the two eyes together and not for each single eye.

**Table 5:** SAR assessment in the connected car.

Study Name	Frequency and Power	EMF Source Position Re: Vehicle Cabin	Quantity Estimated & Main Study Outcomes
Jeladze et al. [32]	450, 900 and 1800 MHz bands at 1 W.	Internal: mobile phone antenna at 2.5 cm from the phantom head.	Maximum point SAR: 96 W/kg at 450 MHz, 296 W/kg at 900 MHz and 127 W/kg at 1800 MHz.
Harris et al. [33]	UMTS (2.1 GHz) at 125–250 mW. WiMax (2.5 GHz) at 200 mW. Bluetooth (2.45 GHz) at 2 mW.	Internal: emitting devices were placed inside the vehicle cabin at driver and passenger seats.	Maximum SAR_wb_: 2.13 mW/kg with UMTS. Maximum SAR_10g_: 25.3 mW/kg with UMTS for head/trunk; 392.6 mW/kg with WiMax for limbs.
Leung et al. [34]	900 MHz band at 2 W.	Internal: mobile phone emitting device placed at user(s)’s ear level.	Maximum SAR_10g_: 3.02 W/kg for single-user condition; 3.77 W/kg for two-user condition.
Tognola et al. [38]	ITS 5.9 GHz at 30 W.	External: four V2V quarter-wave monopole antennas mounted on the roof of the car	Maximum SAR_wb_: 0.008 W/kg. Maximum SAR_10g_: 1.58 W/kg for head/torso; 0.76 W/kg for limbs.
Present study	3.5 GHz band at 1 W.	External: two 5G-V2X array antennas positioned in the rear and front position on the car body	Maximum SAR_wb_: 0.074 mW/kg. Maximum SAR_10g_: 6.818 mW/kg.

## Data Availability

Not applicable.

## References

[1] Sjoberg K., Andres P., Buburuzan T., Brakemeier A. (2017). Cooperative Intelligent Transport Systems in Europe: Current Deployment Status and Outlook. IEEE Veh. Technol. Mag..

[2] Shen X., Fantacci R., Chen S. (2020). Internet of Vehicles [Scanning the Issue]. Proc IEEE..

[3] Wang J., Shao Y., Ge Y., Yu R. (2019). A Survey of Vehicle to Everything (V2X) Testing. Sensors.

[4] Ghosal A., Conti M. (2020). Security issues and challenges in V2X: A Survey. Comput. Netw..

[5] Rebbeck T., Stewart J., Lacour H.A., Lillen A., McClure D., Dunoyer A. (2017). Socio-economic benefits of cellular V2X. Final Report for 5GAA.

[6] (2010). IEEE Standard for Information Technology—Local and Metropolitan Area Networks—Specific Requirements—Part 11: Wireless LAN Medium Access Control (MAC) and Physical Layer (PHY) Specifications Amendment 6: Wireless Access in Vehicular Environments.

[7] (2017). Intelligent Transport Systems (ITS); Radiocommunications Equipment Operating in the 5855 MHz to 5925 MHz Frequency Band; Harmonised Standard Covering the Essential Requirements of Article 3.2 of Directive 2014/53/EU.

[8] (2020). Intelligent Transport Systems (ITS); LTE-V2X Access Layer Specification for Intelligent Transport Systems Operating in the 5 GHz Frequency Band.

[9] 5GAA (2018). Coexistence of C-V2X and ITS-G5 at 5.9GHz. Position-Paper.

[10] Bazzi A., Cecchini G., Menarini M., Masini B.M., Zanella A. (2019). Survey and Perspectives of Vehicular Wi-Fi versus Sidelink Cellular-V2X in the 5G Era. Future Internet.

[11] (2021). Technical Specification Group Radio Access Network; V2X Services Based on NR; User Equipment (UE) Radio Transmission and Reception, Release 16.

[12] (2021). Technical Specification Group Radio Access Network; NR Sidelink Enhancement; User Equipment (UE) Radio Transmission and Reception, Release 17.

[13] 5GPPP (2019). 5GCAR Deliverable D2.1: 5GCAR Scenarios, Use Cases, Requirements and KPIs.

[14] Sreelakshmi K., Bora P., Mudaliar M., Baburao Dhanade Y.B., Madhav B.T.P. (2017). Linear array Yagi-Uda 5G antenna for vehicular application. Int. J. Eng. Technol..

[15] Bamy C.L., Moukanda Mbango F., Konditi D.B.O., Moukala Mpele P. (2021). A compact dual-band Dolly-shaped antenna with parasitic elements for automotive radar and 5G applications. Heliyon.

[16] Yacoub A., Aloi D. (2021). Low Profile PIFA Antenna for Vehicular 5G and DSRC Communication Systems.

[17] Hasturkoglu S., Lindenmeier S. Antenna Module with New Wideband 5G-Antenna Array at 28 GHz in Combination with GNSS- and 4G/WLAN/DSRC in Automotive Environment. Proceedings of the 2018 48th European Microwave Conference (EuMC).

[18] Pfadler A., Ballesteros C., Romeu J., Jofre L. (2020). Hybrid Massive MIMO for Urban V2I: Sub-6 GHz vs mmWave Performance Assessment. IEEE Trans. Veh. Technol..

[19] Kakkavas A., García M.H.C., Stirling-Gallacher R.A., Nossek J.A. Multi-Array 5G V2V Relative Positioning: Performance Bounds. Proceedings of the 2018 IEEE Global Communications Conference (GLOBECOM).

[20] Nasr A., Sarabandi K., Takla M. (2020). Multi-beam Dual-Polarized Windshield Antenna with Wide Elevation Coverage for 5G V2X Applications. Proceedings of the 2020 IEEE International Symposium on Antennas and Propagation and North American Radio Science Meeting.

[21] Bushberg J.T., Chou C.K., Foster K.R., Kavet R., Maxson D.P., Tell R.A., Ziskin M.C. (2020). IEEE Committee on Man and Radiation—COMAR Technical Information Statement: Health and Safety Issues Concerning Exposure of the General Public to Electromagnetic Energy from 5G Wireless Communications Networks. Health Phys..

[22] Dürrenberger G., Fröhlich J., Röösli M., Mattsson M.O. (2014). EMF monitoring—Concepts, activities, gaps and options. Int. J. Environ. Res. Public Health.

[23] Ramírez-Vázquez R., Escobar I., Franco T., Arribas E. (2022). Physical units to report intensity of electromagnetic wave. Environ. Res..

[24] Morelli M.S., Gallucci S., Siervo B., Hartwig V. (2021). Numerical Analysis of Electromagnetic Field Exposure from 5G Mobile Communications at 28 GHZ in Adults and Children Users for Real-World Exposure Scenarios. Int. J. Environ. Res. Public Health.

[25] Bonato M., Dossi L., Chiaramello E., Fiocchi S., Gallucci S., Tognola G., Ravazzani P., Parazzini M. (2021). Human RF-EMF Exposure Assessment Due to Access Point in Incoming 5G Indoor Scenario. IEEE J. Electromagn. RF Microw. Med. Biol..

[26] Bonato M., Dossi L., Gallucci S., Benini M., Tognola G., Parazzini M. (2022). Assessment of Human Exposure Levels Due to Mobile Phone Antennas in 5G Networks. Int. J. Environ. Res. Public Health.

[27] Morimoto R., Hirata A., Laakso I., Ziskin M.C., Foster K.R. (2017). Time constants for temperature elevation in human models exposed to dipole antennas and beams in the frequency range from 1 to 30 GHz. Phys. Med. Biol..

[28] Thors B., Colombi D., Ying Z., Bolin T., Törnevik C. (2016). Exposure to RF EMF From Array Antennas in 5G Mobile Communication Equipment. IEEE Access.

[29] Laakso I., Morimoto R., Heinonen J., Jokela K., Hirata A. (2017). Human exposure to pulsed fields in the frequency range from 6 to 100 GHz. Phys. Med. Biol..

[30] Shikhantsov S., Thielens A., Vermeeren G., Tanghe E., Demeester P., Martens L., Torfs G., Joseph W. (2019). Hybrid Ray-Tracing/FDTD Method for Human Exposure Evaluation of a Massive MIMO Technology in an Industrial Indoor Environment. IEEE Access.

[31] Tognola G., Bonato M., Benini M., Aerts S., Gallucci S., Chiaramello E., Fiocchi S., Parazzini M., Masini B.M., Joseph W. (2022). Exposure to RF Electromagnetic Fields in the Connected Vehicle: Survey of Existing and Forthcoming Scenarios. IEEE Access.

[32] Jeladze V.B., Nozadze T.R., Tabatadze V.A., Petoev-Darsavelidze I.A., Prishvin M.M., Zaridze R.S. (2020). Electromagnetic Exposure Study on a Human Located inside the Car Using the Method of Auxiliary Sources. J. Commun. Technol. Electron..

[33] Harris L.R., Zhadobov M., Chahat N., Sauleau R. (2011). Electromagnetic dosimetry for adult and child models within a car: Multi-exposure scenarios. Int. J. Microw. Wirel. Technol..

[34] Leung S.-W., Diao Y., Chan K.-H., Siu Y.-M., Wu Y. (2012). Specific Absorption Rate Evaluation for Passengers Using Wireless Communication Devices Inside Vehicles with Different Handedness, Passenger Counts, and Seating Locations. IEEE Trans. Biomed. Eng..

[35] Baramili E., Sarkis R., Saleh M.B. (2020). Investigation of Driver EMF Exposure From 4G/5G Automotive Glass Mounted Antennas. Proceedings of the 2020 IEEE International Symposium on Antennas and Propagation and North American Radio Science Meeting.

[36] Ruddle A.R., Low L., Rigelsford J.M., Langley R.J. Variation of computed in-vehicle SAR with number and location of occupants at commonly used communications frequencies. Proceedings of the 10th International Symposium on Electromagnetic Compatibility.

[37] Ruddle A.R. (2016). Preliminary estimates of electromagnetic field exposures due to advanced vehicle technologies. Proceedings of the 2016 Loughborough Antennas & Propagation Conference (LAPC).

[38] Tognola G., Masini B., Gallucci S., Bonato M., Fiocchi S., Chiaramello E., Parazzini E., Ravazzani P. (2021). Numerical Assessment of RF Human Exposure in Smart Mobility Communications. IEEE J. Electromagn. RF Microw. Med. Biol..

[39] Ziegelberger G., Croft R., Feychting M., Green A.C., Hirata A., d’Inzeo G., Jokela K., Loughran S., Marino C., Miller S. (2020). ICNIRP guidelines for limiting exposure to electromagnetic fields (100 kHz to 300 GHz). Health Phys..

[40] Ruddle A.R. Influence of dielectric materials on in-vehicle electromagnetic fields. Proceedings of the 2008 IET Seminar on Electromagnetic Propagation in Structures and Buildings.

[41] H2020 5G PPP 5GCar Project. https://5gcar.eu..

[42] 5GPPP (2018). 5GCAR Deliverable D3.1: Intermediate 5G V2X Radio.

[43] Artner G., Kotterman W., Del Galdo G., Hein M.A. (2019). Automotive Antenna Roof for Cooperative Connected Driving. IEEE Access.

[44] TE Connectivity White Paper (2019). V2X—An Important Building Block in Cooperative Intelligent Transport Systems (C-ITS). https://www.te.com/content/dam/te-com/documents/automotive/global/automotive-next-gen-mobility-v2x-09-2019-en.pdf.

[45] Gosselin M.C., Neufeld E., Moser H., Huber E., Farcito S., Gerber L., Jedensjö M., Hilber I., Gennaro F.D., Lloyd B. (2014). Development of a new generation of high-resolution anatomical models for medical device evaluation: The Virtual Population 3.0. Phys. Med. Biol..

[46] Gabriel C., Gabriel S., Corthout E. (1996). The dielectric properties of biological tissues: I. Literature survey. Phys. Med. Biol..

[47] Gabriel S., Lau R.W., Gabriel C. (1996). The dielectric properties of biological tissues: II. Measurements in the frequency range 10 Hz to 20 GHz. Phys. Med. Biol..

[48] Taflove A., Hagness S.C., Piket-May M. (2004). Computational electromagnetics: The finite-difference time-domain method. The Electrical Engineering Handbook.

[49] Uusitupa T., Laakso I., Ilvonen S., Nikoskinen K. (2010). SAR variation study from 300 to 5000 MHz for 15 voxel models including different postures. Phys. Med. Biol..

[50] Bonato M., Dossi L., Chiaramello E., Fiocchi S., Tognola G., Parazzini M. (2021). Stochastic Dosimetry Assessment of the Human RF-EMF Exposure to 3D Beamforming Antennas in indoor 5G Networks. Appl. Sci..

[51] Tognola G., Bonato M., Chiaramello E., Fiocchi S., Magne I., Souques M., Parazzini M., Ravazzani P. (2019). Use of Machine Learning in the Analysis of Indoor ELF MF Exposure in Children. Int. J. Environ. Res. Public Health.

